# Introducing Three New Fruit-Scented Mints to Farmlands: Insights on Drug Yield, Essential-Oil Quality, and Antioxidant Properties

**DOI:** 10.3390/antiox11050866

**Published:** 2022-04-28

**Authors:** Hosein Ahmadi, Mohammad Reza Morshedloo, Roya Emrahi, Abdollah Javanmard, Farzad Rasouli, Filippo Maggi, Manoj Kumar, Jose Manuel Lorenzo

**Affiliations:** 1 Department of Horticulture Science, College of Agriculture and Natural Resources, University of Tehran, Karaj P.O. Box 31587 77871, Iran; hoseinahmadi@ut.ac.ir; 2 Department of Horticultural Science, Faculty of Agriculture, University of Maragheh, Maragheh P.O. Box 83111 55181, Iran; royaemrahi@maragheh.ac (R.E.); irfarzad.rasouli@maragheh.ac.ir (F.R.); 3 Department of Plant Production and Genetics, Faculty of Agriculture, University of Maragheh, Maragheh P.O. Box 83111 55181, Iran; a.javanmard@maragheh.ac.ir; 4 Chemistry Interdisciplinary Project (ChIP), School of Pharmacy, University of Camerino, 62032 Camerino, Italy; filippo.maggi@unicam.it; 5 Chemical and Biochemical Processing Division, ICAR–Central Institute for Research on Cotton Technology, Mumbai 400019, India; manoj.kumar13@icar.gov.in; 6 Centro Tecnológico de la Carne de Galicia, Avda. Galicia Nº 4, Parque Tecnológico de Galicia, San Cibrao das Viñas, 32900 Ourense, Spain; 7 Área de Tecnoloxía dos Alimentos, Facultade de Ciencias, Universidade de Vigo, 32004 Ourense, Spain

**Keywords:** mint species, essential-oil yield, dry-weight yield, linalool, linalyl acetate, piperitone oxide, menthol

## Abstract

Mint species are one of the most traded medicinal plants with a wide array of applications in the food, pharmaceutical, and perfumery industries. Here, a field experiment based on completely randomized block design (RCBD) aimed to compare drug yield, antioxidant properties, and essential-oil (EO) quality of three newly introduced mints (i.e., ginger mint, pineapple mint, and grapefruit mint) with a chiefly cultivated one (i.e., peppermint). The results manifested that dry-weight yield and EO yield of grapefruit mint (310 g/m^2^ and 5.18 g/m^2^, respectively) was approximately 2 times more than that of others. The highest EO content (i.e., 3.12%, *v*/*w*)) was obtained from the ginger mint; however, there were no significant differences among the other three mints. The highest total flavonoids content and 2,2-diphenyl-1-picrylhydrazyl (DPPH) scavenging activity of both methanolic and ethanolic extracts were found in pineapple and grapefruit mint. Methanolic extract of ginger mint yielded the highest total polyphenol content, whereas the ethanolic extract of pineapple mint showed the highest total polyphenol content. According to mean comparisons, the EO of ginger mint exhibited the highest antioxidant activity (EC_50_ value = 2.23 µL/mL), while EO of peppermint showed the lowest antioxidant activity (EC_50_ value = 48.23 µL/mL). Gas chromatography analysis identified four EO types among these mints: (i) grapefruit mint EO rich in linalool (51.7%) and linalyl acetate (28.38%); (ii) ginger mint EO rich in linalool (59.16%); (iii) pineapple mint EO rich in piperitone oxide (77.65%); and (iv) peppermint EO rich in menthol (35.65%). The findings of the present study provide new insights into the cultivation of preferable mints possessing desired characteristics for food and drug industries.

## 1. Introduction

The genus *Mentha* (Lamiaceae) comprises over 60 species and is widely distributed throughout the world, especially in temperate and semi-temperate zones [1]. The aroma profile, determined by the essential-oil chemical profile, is one of the most important discriminative in mint quality evaluation. Interspecific variation is the most affecting factor on the yield and quality of mint essential oils [2]. For their distinct aroma and flavors, several cultivars and species of mint have already been used in food commodities such as confectionery, chewing gums, cheese, soups, salads, and herbal teas [3]. Moreover, systematic scientific evidence has already corroborated the effectiveness of herbal preparations from mint species against digestive disorders, fever, spasm, and inflammation [4,5]. Many researchers have constantly mentioned several therapeutic benefits of mint species such as antioxidant, antimicrobial, antihypertensive, antiallergic, and sedative activity, which is probably linked to the presence of polyphenolic constituents and terpenoids [4,6]. Owing to functional OH groups, polyphenols, flavonoids, and some terpenoid compounds act as natural antioxidants in the human body, negating the deleterious detriment of free radicals that usually overwhelm protective enzymes and trigger deteriorative cellular effects [7,8]. Essential oils (EOs) are economically important natural products with a wide range of applications in downstream industries [9]. EOs and extracts from mint species are frequently applied as natural ingredients in herbal remedies and cosmetic preparations [5]. The application of EOs in food commodities (instead of synthetic products with potentially proven harmful effects such as butylated hydroxyanisole (BHA) and butylated hydroxytoluene (BHT)) can serve as an authentic alternative to prevent oxidative damages and elongate the lifespan [10].

As a result of interspecific hybridization, the genus *Mentha* shows a vast range of morphological and phytochemical variability [11]. Interspecific hybridization, whether occurring naturally or artificially, makes it possible to acquire cultivars with desired flavor, aroma, and appearance [11]. Tucker et al. [12,13] reported that ginger mint (*M.* x *gracilis)*, commonly known as scotch spearmint, is a naturally occurring sterile hybrid resulting from the cross between spearmint (*M. spicata*) and corn mint (*M. arvensis*). Ginger mint is indigenous to Europe and Asia and has elegant bright yellow stripes on the leaves [12]. In addition to its medicinal properties, the variegated ginger mint is also cultivated for its ornamental features [14]. Due to its exhilarating sweet odor, the plant is typically used in the liquor, herbal beverages, and confectionery industries [14]. Grapefruit mint (*M. suaveolens × piperita*) is another sterile hybrid resulting from the cross between *M. suaveolens* and *M × piperita* [15]. The perennial herb grows properly when adapted to adequate sunlight [16]. Grapefruit mint releases a strong aroma of citrus fruits and is often used in herbal tea, juices, desserts, salads, and cooking [17]. Medical investigations have substantiated that polar compounds in the aqueous extract of grapefruit mint are responsible for its anticytopathogenicity properties and human immunodeficiency virus (HIV-1) reverse-transcriptase-suppressing activity [18]. The essential oil of grapefruit mint also has great potential for applications in bath and oral hygiene products, syrups, and ice cream [19]. Pineapple mint is one of the variegated cultivars (cv. *variegata*) of *M. suaveolens* possessing bumpy and hairy leaves usually surrounded with white margins [20]. Pineapple mint is often planted as an ornamental plant, fragrant groundcover, and can also grow in pots and hanging baskets [16]. The intoxicating citrus scent of its leaves makes the plant an ideal choice for use in soft drinks, infusions, and aromatherapy in order to improve digestion and eliminate fatigue [16].

By the end of 2022, the production of essential oils is expected to reach a turnover of more than USD 27 billion, and according to statistics, EOs of mint species are among the top 10 most traded essential-oil products (www.statista.com, accessed on 8 April 2022).

To the best of our knowledge, there are no comparative reports on agronomic yield, drug yield, and aroma profile of the above-mentioned mints. In this respect, the present study mainly aimed to evaluate the essential-oil yield and distinguish odor-determining compositions as well as antioxidant activity of three newly introduced mints to Iranian farmlands. This study herein provides a reliable source for the acceptability of these aromatic plants and aids in the breeding of mint species with desired aroma and flavor.

## 2. Materials and Methods

### 2.1. Cultivation and Growth Condition

The experiment was carried out during the 2019 growing season in the research field of Maragheh University, Maragheh, Iran. The meteorological data (monthly average temperature and total monthly precipitation of the experimental site) are mentioned in Table 1.

The soil’s physical and chemical characteristics, as well as minerals, are listed in Table 2. Before planting, the soil was plowed and mixed with sufficient manure (200 kg 100 m^−2^) to add organic nitrogen and minerals. Then rhizomes of four mints as four treatments were clonally propagated and cultivated in a completely randomized block design (RCBD) with three replicates (three blocks). In the field, 12 plots (four plots in each block) were arranged and there were five rows in each plot with a length of 3 m. The distances between rows and seedlings of the planting line were considered 50 and 30 cm, respectively. Totally, 50 seedlings were planted per plot (i.e., seven plants per m^2^). During the entire experiment, no chemical fertilizer or pesticide was used, and weeds were controlled every day to support the good growth of mint seedlings. During the growth stage, plants were irrigated every three days using a drip irrigation system. Finally, full-blossoming plants of each plot (50 plants) were evenly cut above the surface and were dried in the oven at 40 °C. Then, the mean values of 50 plants’ dry weight per plot were recorded and divided by the cultivated area to calculate dry-weight yield (g m^−2^).

### 2.2. Essential-Oil Extraction

For essential-oil extraction, harvested plants from each plot were pooled together and then ground using an electrical grinder. After that, 100 g of ground dry materials from each plot were randomly selected and distilled for 3 h using a Clevenger apparatus. The volume of EO was read through the graded burette of Clevenger, and its value (%) was obtained by calculating the portion of EO volume to dry weight of the samples (*v*/*w*). The extracted EOs were weighted using a sensitive scale and then the values were divided by the cultivated area of each replicate per treatment to calculate the EO yield (g/m^−2^).

### 2.3. Alcoholic Extractions and Antioxidant Properties

Hydroalcoholic extraction and subsequent assays were carried out using the method described by Ahmadi et al. [9] with slight modifications. Accordingly, 1 g of dried leaves was added into 40 mL of methanol 80% (in water) and 40 mL of ethanol 70% (in water). The procedure was continued by shaking samples at 100 rpm for 24 h. After centrifugation, the supernatants were collected in new tubes and the residuals were re-extracted in the second step for another 24 h.

Total polyphenol contents of extracts were determined using 10-times-diluted Folin-Ciocalteu’s reagent and 3% solution of NaHCO_3_. Then, the absorbance of samples was read at 765 nm. The total content of polyphenols was expressed in terms of mg gallic acid equivalent (GAE) g^−1^ dried weight.

Total amounts of flavonoids in the hydroalcoholic extracts were estimated using AlCl_3_ reagent. The absorbance of the investigated samples was read at 415 nm using a microplate reader and quantification was carried out when the calibration curve of quercetin was created. The total content of flavonoids was expressed in terms of mg quercetin equivalent (QE) g^−1^ dried weight.

The method explained in our previous study was applied to determine the 2,2-diphenyl-1-picrylhyrazyl (DPPH) free-radical scavenging activity of extracts. In this assay, hydroalcoholic extracts of mint leaves reacted with 0.2 mM methanolic solution of DPPH for 30 min under dark conditions. The absorbance of samples was recorded at 517 nm. The value of the measurement was expressed in terms of percentage inhibition of DPPH free-radical and calculated using Equation (1).

%I = 100 × (Absorbance blank − Absorbance sample)/Absorbance blank(1)

### 2.4. DPPH Scavenging Activity of Essential Oils

The DPPH assay was conducted according to the outlines described by Morshedloo et al. [21] with minor modification. For this, 10 µL of diluted series (0.5–50 µL/mL) of the essential oils in methanol were vigorously mixed with 1 mL of a 0.2 mM methanol solution of DPPH. After incubation for 30 min, the absorbance of 300 µL of the samples was read at 517 nm using a Bio-Tek microplate reader. Then, linear regression helped in estimating half-maximal effective concentration (EC_50_) values.

### 2.5. Profiling and Quantification of Volatile Constituents

The identification and quantification procedure was carried out according to the method explained by our previous study [7]. GC–MS analysis was performed using an Agilent 7990B gas chromatograph coupled to a mass spectrometer (5977A, Agilent, Santa Clara, CA, USA). The mentioned device was equipped with an HP-5MS capillary column (5% phenylmethyl polysiloxane, 30 m length, 0.25 mm internal diameter, and 0.1 μm film thickness). The gradient temperature program was set up as follows: 5 min at 60 °C, subsequently 3 °C min^−1^ to 230 °C. Helium was used as carrier gas with a flow rate of 1 mL min^−1^. Injector and transfer line temperatures were set at 230 and 240 °C, respectively. The split ratio of the injector was 1:30 and the mass scan ranged from 40 to 450 *m/z*. To identify volatile constituents, a complementary procedure was executed including calculation of arithmetic retention indices regarding the coherence of homologous series of hydrocarbons (Supelco, Bellefonte, PA, USA), comparing retention indices with those reported in the reference literature [22], and browsing mass-acquisition data in the WILEY275 and NIST 08 libraries. As an additional procedure, some compounds were validated by comparing their retention indices with those of authentic standards (Supelco phytochemical standards, Bellefonte, PA, USA).

Gas chromatography (GC) analysis coupled with a flame ionization detector (FID) was carried out using an Agilent 7990B instrument. GC-FID device possessed VF- 5MS column which had the same stationary phase and dimensions as the HP-5MS one. Moreover, the same thermal program described above was applied for GC-FID analysis. For the quantification process, internal peak areas of each essential-oil composition were automatically integrated. The peak areas were normalized without using correction factors. Before injection, the EO samples were first diluted with *n*-hexane (1:100) and then 1 μL aliquot was used for analysis. The relative amount of each compound was expressed as a proportional percentage of the constituent.

### 2.6. Statistical Analysis

Analysis of variance (ANOVA) based on randomized complete block design (RCBD) and mean comparisons (least significant differences (LSD) test) were performed using SAS 9.4 software (SAS Institute, Cary, NC, USA). The multivariate analysis was also conducted through Xlstat 2019 software (Microsoft, Redmond, WA, USA).

## 3. Results and Discussion

### 3.1. Dry-Weight Yield and Essential-Oil Productivity

**Figure 1:**
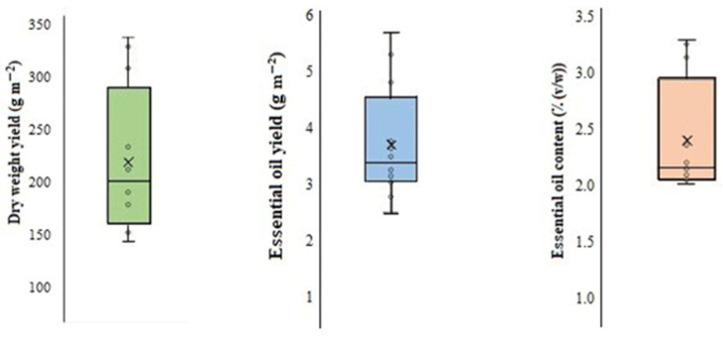
Box plot representing yield traits of mint species (*n* = 12).

**Figure 2:**
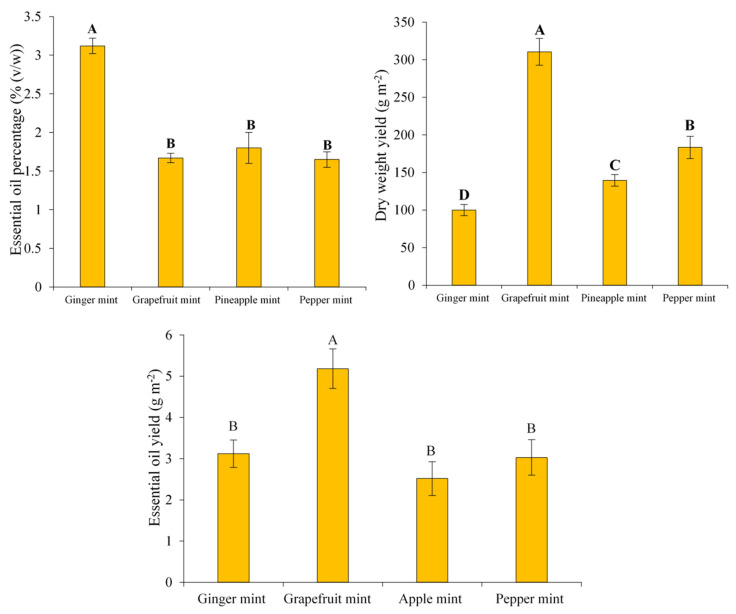
Mean comparison of yield-related parameters of the four mint species (values + or − standard deviation (*n* = 3)). Means with same letter does not have significant difference.

ANOVA displayed that there were significant differences (*p* < 0.01) among mints in terms of dry-weight yield, EO yield, and EO percentage. According to the boxplot presented in Figure 1, a wide range of variability was seen among mints in terms of dry-weight yield (91.6–310 g/m^2^), EO percentage (1.55–3.12%), and EO yield (2.146–5.63 g/m^2^). According to mean comparisons, grapefruit mint yielded the highest dry weight per m^2^ (310 g/m^2^); thereafter, peppermint (183.3 g/m^2^), pineapple mint (139.4 g/m^2^), and ginger mint (99.97 g/m^2^) were in the next orders (Figure 2). Although it was observed that ginger mint contained more essential oil (3.12%, *v*/*w*), the plant yielded the lowest dry weight (100 g/m^2^) and thus showed a lower drug yield (i.e., EO yield) than grapefruit mint on the whole (Figure 2). There were no significant differences among the other three mints in terms of EO content (Figure 2). In agreement with the present study, a comparative study demonstrated that ginger mint possesses more essential oil content than apple and pineapple mints [20]. The range of EO yield reported in a previous study is close to that of our investigation [14]. Indeed, many factors such as expression of growth-related genes, soil conditions, water availability, and light intensity lie on species-specific differences in terms of agronomic yield, EO quantity, and amounts of valuable compounds in medicinal plants [23].

Concerning the establishment of the same environmental and growing conditions, such differences in yield traits may be linked to the genetic backgrounds of these mints. Since natural interspecific hybridization occurs abundantly among mint species, genetic breeding in *Mentha* genus can potentially aid in achieving superior agronomic properties, high yielding cultivars, EOs richness, and improved quality of EOs with desired compositions which eventually will end in better economic efficiency of mint-related commodities [23].

### 3.2. Antioxidant Properties

**Figure 3:**
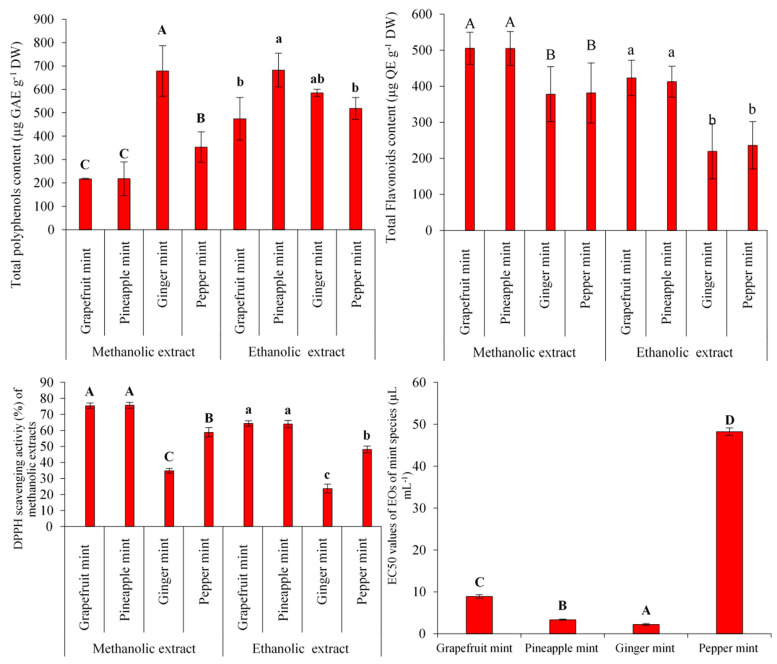
Mean comparison of total polyphenols, total flavonoids, and antioxidant properties of the four mint species (values + or − Standard deviation (*n* = 3)). Means with same letters does not have significant difference.

According to the analysis of variance, significant differences (*p* < 0.01) were found among the four mints in terms of total polyphenols content, total flavonoids, and antioxidant activity of hydroalcoholic extracts and essential oils. On the whole, the use of ethanol showed the highest efficiency to extract phenols, whereas the methanolic extract yielded the highest flavonoids content and DPPH radical scavenging activity in all samples (Figure 3).

**Figure 4:**
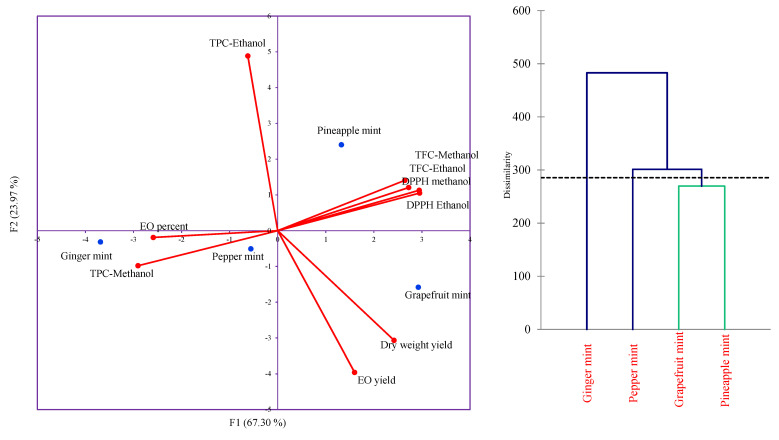
Agglomerative hierarchical clustering and principal component analysis (PCA) of measured traits.

It can be inferred in this sense that extraction efficiencies of phenols are diminished with the enhancement of solvent polarity. The retrieval of antioxidant polyphenols in different kinds of solvents is highly influenced by the degree of solvent polarity and the solubility of these compounds [24,25]. From the usage of completely polar solvents such as water, only a small amount of low-polar polyphenolic compounds will enter the extract solution [26]. On the other hand, it is well-demonstrated that the antioxidant activity of extracts is dependent on several factors such as concentration of extract, temperature, the abundance of hydrophobic or amphipathic compounds, synergistic effects, and chemical nature of the solvent used to extract the herbal materials [27]. Overall, pineapple and grapefruit mint showed the highest total flavonoid content and DPPH scavenging activity in both methanolic (505 µg QE/g dried weight and 75%, respectively) and ethanolic (423 and 412 µg QE/g dried weight and 64%, respectively) extracts; however, there were no statistically significant differences between them (Figure 3). According to Figure 3, methanolic extract of ginger mint yielded the highest total polyphenol content (678.7 µg GAE/g dried weight), whereas the ethanolic extract of pineapple mint showed the highest total polyphenol content (682.6 µg GAE/g dried weight). Flavonoids and phenolic compounds not only play functional roles in plants kingdom such as protecting from UV rays, conferring tolerance against environmental stresses, activating signaling networks, and protecting against pathogens, but also are used as naturally strong antioxidants in promoting human health and suppressing oxidative stress, mainly due to their functional OH groups [24,25]. As illustrated in Figure 4, agglomerative hierarchical clustering (UPGMA method) and principal component analysis (PCA) indicated that the extracts from grapefruit and pineapple mint possess the highest antioxidant properties, which makes them appropriate choices for pharmaceutical products based on herbal extracts (soaps, tinctures, herbal teas, and syrups). Moreover, mean comparisons presented in Figure 3 demonstrate that the EO of ginger mint possesses the highest antioxidant activity (EC_50_ value = 2.23 µL/mL), while the EO of peppermint has the lowest antioxidant activity (EC_50_ value = 48.23 µL/mL). Application of EOs will donate natural antioxidant agents to food manufactures instead of using synthetic ones and this feature is the precedence of ginger mint among the so-called fruit-scented mint species [10].

**Figure 5:**
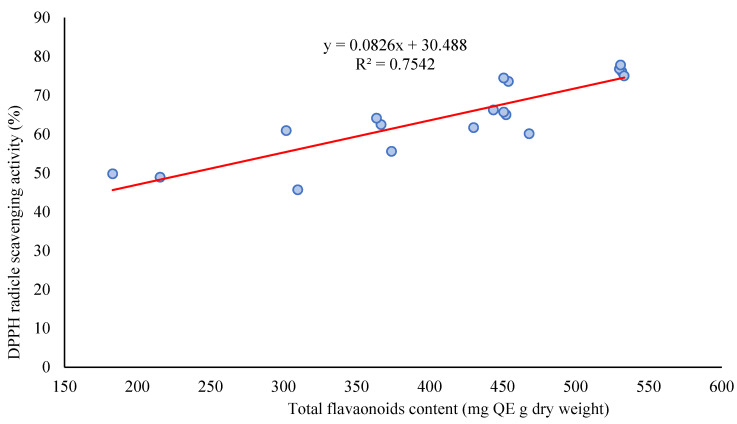
The relationship between DPPH radical scavenging activity and total flavonoids content.

Notably, there was found an interesting positive correlation between total flavonoids content and the DPPH radical scavenging activity (r = 0.86, *p* < 0.01) according to linear regression analysis (Figure 5).

### 3.3. Profile of Volatile Constituents

The variations of EO profile among the four mints were examined through GC-FID and GC-MS analysis. GC–MS analysis identified a total of 44 constituents in the EOs of the four mints (Table 3). Oxygenated monoterpenes were the most abundant class of terpenes (>65%). The major compounds in EOs of ginger mint and pineapple mint were linalool (51.7%) and piperitenone oxide (77.65%), respectively. According to the report from Tsuneya et al. [28], eugenol was the major compound of ginger mint EO. In another study, carvone and limonene were present as the main component of *M. gracilis* EO [14]. Wang et al. [16] reported that germacrene D was the major compound of pineapple mint EO. In the current study, grapefruit mint EO was rich in linalool (51.7%) and linalyl acetate (28.38%). Menthol (35.65%) and menthone (26.81%) characterized the EO of peppermint. Inconsistent with the report by Wang et al. [19], linalool (41.50%) and linalyl anthranilate (33.75%) were present as main components in the EO of grapefruit mint. In a recent paper, three groups of chemotypes (*trans*-piperitenone oxide, carvone or menthol, and related compounds), were characterized among fifteen mint cultivars from four species (*M. arvensis, M.* × *piperita, M. suaveolens* and *M. spicata*) according to the abundance of these constituents in the EO profile [11]. The mentioned study well-concurred with the findings of the present report in indicating piperitenone oxide as the major compound of pineapple mint EO [16]. Combination of sensory, spectroscopic and chemometric measurements allowed to discriminate potential odor-active markers such as *α*-citral, menthofuran, *iso*-menthone, menthol, carvone, and linalool among the five mints. Linalool was characterized by GC-olfactometric-MS analysis releasing the scent of citrus fruits and floral perfume [2]. In conclusion, the identification of three scent-determining monoterpenes (piperitenone oxide, linalool, linalyl acetate) in the essential oils of three newly introduced mints broadens knowledge for their future applications in the food and perfumery industries of Iran and other countries.

## 4. Conclusions

Four key indices bear directly on the drug yield of an aromatic medicinal plant, i.e., dry-weight production, the ratio of economically important organs, total essential-oil content, and relative amount of its major compounds. The present study concluded that there is a substantial difference in drug yield, chemical composition, and antioxidant properties of four so-called fruit-scented mints. In this regard, we were witnessed that essential-oil yield among mints shows a wide range of variation (2.14–5.63 g/m^2^). Since grapefruit mint produced more dry weight and gave more essential oil content, it can be cultivated as a new alternative to achieving a higher yield per m^2^/hectare. Although ginger mint yielded more essential-oil content (%), the plant’s very low dry weight (100 g/m^2^) caused a diminished total yield. However, the essential oil of ginger mint possessed the highest antioxidant activity, which makes its essential oil an ideal flavoring and conservative agent in food industries. On the whole, the results of the mean comparison demonstrated that antioxidant properties of both methanolic and ethanolic extracts in grapefruit and pineapple mint were higher than those of the two other mints, making them appropriate options for pharmaceutical purposes (tinctures, herbal teas, and syrups). Furthermore, the identification of three chemotypes among introduced mints (each one with a distinct aroma and flavor) widens their scent-dependent applications in food and perfumery industries.

## Figures and Tables

**Table 1:** Monthly average temperature (°C) and total monthly precipitation (mm) in 2019 harvest year.

Month	Monthly Average Temperature		Total Monthly Precipitation	
	2019	2 Year Mean	2019	2 Year Mean
April	10.44	11.53	51.34	48.14
May	18.54	17.52	37.80	46.18
June	25.74	24.89	4.2	2.96
July	27.62	28.92	0	0.05
August	27.83	27.75	0.03	0.01
September	22.15	22.84	0	0.11
October	16.64	16.30	6.32	12.79

**Table 2:** Soil analysis results before initiating the experiment (depth 0–30 cm).

Texture	Sand (%)	Silt (%)	Clay (%)	Organic Matter (%)	EC (dS/m)	(pH)	Amount of Exchangeable	Cation Exchange Capacity (Cmolc/kg)	Available Phosphorus (mg/kg)	Total Nitrogen (%)
(Sandy clay loam)	55	16.4	27.4	1.22	1.17	8.15	570.74	26	9.41	0.088

**Table 3:** The relative peak area percentages of essential-oil compositions (Values ± Standard deviation) analyzed in four odorous (*n* = 3) species of mint.

*n*	RI Calc ^a^	RI Lit ^b^	ID ^c^	Compound	Grapefruit Mint (*M. suaveolens* × *M. piperita*)	Ginger Mint (*Mentha* × *gracilis*)	Pineapple Mint (*Mentha suaveolens* var. *variegata*)	Peppermint (*Mentha* × *piperita*)
1	923	924	RI-MS	*α*-Thujene	-	1.03 ± 0.15	-	-
2	929	930	RI-MS-std	Citronellene	0.04 ± 0.01	-	0.51 ± 0.05	-
3	932	932	RI-MS	*α*-Pinene	-	0.82 ± 0.14	-	0.51 ± 0.07
4	969	969	RI-MS	Sabinene	-	-	0.29 ± 0.03	0.54 ± 0.06
5	972	974	RI-MS	*β*-Pinene	0.14 ± 0.06	5.81 ± 0.14	0.66 ±0.06	0.88 ± 0.08
6	988	988	RI-MS-std	*β*-Myrcene	0.93 ± 0.16	1.42 ± 0.16	0.52 ± 0.04	0.35 ± 0.04
7	1001	1002	RI-MS	*α*-Phellandrene	-	0.02 ± 0.01	-	-
8	1013	1014	RI-MS-std	*α*-Terpinene	-	0.50 ± 0.23	-	0.3 ± 0.04
9	1026	1024	RI-MS	Limonene	-	-	-	2.38 ± 0.35
10	1021	1024	RI-MS	*p*-Cymene	0.32 ± 0.13	5.65 ± 0.52	-	-
11	1025	1025	RI-MS-std	Limonene	0.81 ± 0.14	-	2.67 ± 0.21	-
12	1027	1026	RI-MS-std	1,8-Cineole	0.78 ± 0.19	2.88 ± 0.36	-	6.99 ± 0.37
13	1036	1032	RI-MS	*(Z)-β*-Ocimene	0.38 ± 0.14	1.03 ± 0.12	0.18 ± 0.01	-
14	1045	1044	RI-MS	*(E)-β*-Ocimene	-	0.25 ± 0.05	-	-
15	1055	1054	RI-MS-std	*γ*-Terpinene	-	4.86 ± 0.30	-	-
16	1066	1065	RI-MS-std	*cis*-Sabinene hydrate	-	-	-	0.95 ± 0.3
17	1085	1084	RI-MS	*trans*-Linalool oxide	-	0.38 ± 0.45	-	-
18	1103	1096	RI-MS-std	Linalool	**51.7 ± 0.68**	**59.16 ± 1.80**	-	0.66 ± 0.03
19	1152	1148	RI-MS-std	Menthone	-	-	-	**26.81 ± 2.59**
20	1161	1159	RI-MS	Menthofuran	-	-	-	2.02 ± 0.37
21	1162	1162	RI-MS	*δ*-Terpineol	-	-	-	3.67 ± 0.31
22	1163	1161	RI-MS	*neo*-Menthol	-	-	-	2.66 ± 0.19
23	1160	1165	RI-MS	Borneol	-	0.03 ± 0.01	0.15 ± 0.08	-
24	1175	1167	RI-MS-std	Menthol	-	-	-	**35.65 ± 0.37**
25	1168	1172	RI-MS	*iso*-Pinocamphone	0.25 ± 0.03	-	-	-
26	1176	1177	RI-MS	Terpinen-4-ol	-	-	-	0.9 ± 0.02
27	1186	1186	RI-MS	*α*-Terpineol	4.92 ± 0.4	1.15 ± 0.15	-	-
28	1225	1227	RI-MS	Nerol	0.91 ± 0.11	-	-	-
29	1235	1233	RI-MS-std	Pulegone	-	-	-	1.09 ± 0.37
30	1252	1252	RI-MS	Piperitone	-	-	-	0.67 ± 0.05
31	1257	1254	RI-MS	Linalool acetate	**28.38 ± 1.50**	-	-	-
32	1291	1289	RI-MS-std	Thymol	1.87 ± 0.67	4.11 ± 0.20	-	-
33	1299	1298	RI-MS-std	Carvacrol	0.91 ± 0.06	0.33 ± 0.10	-	-
34	1363	1361	RI-MS	Neryl acetate	1.24 ± 0.17	-	-	-
35	1366	1366	RI-MS	Piperitenone oxide	-	1.56 ± 0.64	**77.65 ± 1.65**	-
36	1382	1381	RI-MS	Geranyl acetate	2.79 ± 0.36	-	-	0.43 ± 0.15
37	1380	1387	RI-MS	*β*-Bourbonene	-	-	0.23 ± 0.02	-
38	1388	1389	RI-MS	*β*-Elemene	-	-	0.36 ± 0.06	-
39	1394	1392	RI-MS	*(Z)*-Jasmone	-	-	0.92 ± 0.21	-
40	1414	1417	RI-MS-std	*trans*-Caryophyllene	-	3.63 ± 0.62	0.51 ± 0.05	-
41	1455	1454	RI-MS	*(E)-β*-Farnesene	0.44 ± 0.18	-	0.95 ± 0.13	0.35 ± 0.15
42	1476	1481	RI-MS	Germacrene D	0.74 ± 0.17	-	6.17 ± 0.85	2.54 ± 0.76
43	1493	1492	RI-MS	Elixene	-	-	-	0.46 ± 0.14
44	1585	1592	RI-MS	Viridiflorol	-	-	1.58 ± 0.24	0.74 ± 0.11
				Monoterpene hydrocarbons	3.4	24.27	4.83	12.9
				Oxygenated monoterpenes	88.94	66.72	77.8	73.04
				Sesquiterpene hydrocarbons	1.18	3.63	9.8	4.09
				Oxygenated sesquiterpenes	-	-	1.58	0.74
				Total	97.57	94.62	95.93	92.55

Bold values show the main constituents of the essential oil. ^a^ Linear retention index on HP-5MS column, experimentally determined using homologous series of C8-C40 alkanes (Sigma-Aldrich, Cary, NC, USA). ^b^ Linear retention index from Adams (2007) and NIST 08 (2008). ^c^ Identification methods: RI, based on comparison of calculated RI with those reported in Adams 2017 library; based on mass spectrometry data and browsing in WILEY, ADAMS and NIST 08 MS databases; std, based on comparison of Retention time (RT), Retention indice (RI), and Mass spectrometer (MS) data with that of authentic compounds.

## Data Availability

The datasets used and/or analyzed during the current study are available from the corresponding author on reasonable request.
